# Molecular mechanisms of skin wound healing in non-diabetic and diabetic mice in excision and pressure experimental wounds

**DOI:** 10.1007/s00441-022-03624-x

**Published:** 2022-04-07

**Authors:** Vito Antonio Baldassarro, Luca Lorenzini, Alessandro Giuliani, Maura Cescatti, Giuseppe Alastra, Micaela Pannella, Bruno Pietro Imbimbo, Gino Villetti, Laura Calzà, Luciana Giardino

**Affiliations:** 1 Interdepartmental Center for Industrial Research in Life Sciences and Technologies (CIRI-SDV), University of Bologna, Bologna, Italy; 2 Department of Veterinary Medical Science (DIMEVET), University of Bologna, Via Tolara di Sopra 41/E Ozzano Emilia, Bologna, 40064 Italy; 3 IRET Foundation, Ozzano Emilia, Bologna, Italy; 4 Chiesi Farmaceutici, Parma, Italy; 5 Department of Pharmacy and Biotechnology (FaBiT), University of Bologna, Bologna, Italy; 6 Montecatone Rehabilitation Institute, Montecatone, Imola, Italy

**Keywords:** Wound healing, Ulcers, Diabetes, Animal models, Hypoxia/ischemia

## Abstract

Experimental models for chronic skin lesions are excision and pressure ulcer, defined as “open” and “closed” lesions, respectively, only the latter characterized by tissue hypoxia. Moreover, systemic diseases, such as diabetes mellitus, affect wound repair. Thus, models for testing new therapies should be carefully selected according to the expected targets. In this study, we present an extensive and comparative histological, immunohistochemical, and molecular characterization of these two lesions in diabetic (db/db) and non-diabetic (C57BL/6 J) mice. In db/db mice, we found significant reduction in PGP9.5-IR innervation, reduction of capillary network, and reduced expression of NGF receptors. We found an increase in VEGF receptor *Kdr* expression, and the PI3K-Akt signaling pathway at the core of the altered molecular network. Db/db mice with pressure ulcers showed an impairment in the molecular regulation of hypoxia-related genes (*Hif1a*, *Flt1*, and *Kdr*), while extracellular matrix encoding genes (*Itgb*3, *Timp1*, *Fn1*, *Col4a1*) were upregulated by hyperglycemia and lesions. Overall, the molecular analysis suggests that db/db mice have a longer inflammatory phase of the wound repair process, delaying the progression toward the proliferation and remodeling phases.

**Supplementary information:**

The online version contains supplementary material available at 10.1007/s00441-022-03624-x.

## Introduction

Wounds of the skin can still represent a major medical problem in a type of injury known as “chronic non-healing wounds” (Han and Ceilley 2017). These wounds usually occur in the presence of systemic diseases or local tissue alterations, such as diabetes, vasculitis, or infectious diseases, but can also occur as a complication of surgical wounds and in elderly bedridden individuals (Jaul et al. 2018). In the USA, chronic wounds affect approximately 6.5 million patients, costing $25 billion per year (Martinengo et al. 2019).

A specific subset of chronic non-healing wounds occurs in diabetic patients, where their incidence is fairly high, and each individual non-healing ulcer is estimated to cost nearly US$50,000 (Raghav 2018). It is also estimated that 2% of diabetic patients in developed countries suffer from non-healing injuries (European Wound Management Agency), while complications such as infections cause 90% of limb amputations (Rodrigues et al. 2016). The incidence of chronic wounds is expected to increase due to the increasing age of the population, and to the worldwide increase in diabetes prevalence, currently four times higher than all cancers combined, contributing to 9% of global mortality (Shaw et al. 2010). Current treatments include wound care with a number of dressings, negative pressure therapy, and hyperbaric oxygen (Han and Ceilley 2017), but only three FDA–approved therapies are available, two of which are skin substitutes and the other the recombinant human platelet-derived growth factor (PDGF-BB, becaplermin, Regranex^®^).

Skin wound healing is a complex process, involving a coordinated sequence of cellular and molecular events (Rodrigues et al. 2016). The process consists of four phases: coagulation, inflammation, proliferation, and remodeling. Systemic disease such as diabetes may impair each of these steps by altering the anatomy and biology of the skin, as well as affecting physiological turnover such as repair attempts (Lima et al. 2017). Hyperglycemia directly and indirectly affects the biology of skin cells (Lee et al. 2019; Gostynska et al. 2020), creates vascular dysfunction and angiopathy (Okonkwo et al. 2020), and alters skin innervation, leading to a small fiber neuropathy (Zilliox et al. 2015), and making the skin more prone to accidental lesions and more difficult to repair.

A variety of skin injuries can lead to ulcer formation, such as accidental or surgical penetrating lesions, repeated tissue pressure and ischemia, or skin infection. A variable common to many causes of skin lesion appears to be tissue hypoxia, resulting from local or systemic diseases (Patel et al. 2019). In fact, more than 80% of chronic wounds are associated with diabetes mellitus, vascular insufficiency, and high blood pressure, all conditions in which capillary dysfunction and tissue hypoperfusion are documented (Masson-Meyers et al. 2020; Okonkwo et al. 2020).

In light of the above, disease models for testing novel therapies should be carefully selected and evaluated depending on the expected molecular and cellular targets and their respective translational potential. “Open” and “closed” lesions can be experimentally induced in different animal species, open lesions usually being induced by skin incision or excision (by skin biopsy needle) to mimic surgical or abrasive insults, while closed lesions are generally induced by experimental ischemia/reperfusion cycles in the vascular plexus in the dermis, mimicking pressure-related ischemia such as bedsores or diabetic feet.

Full-thickness skin excision and repeated hypoxia/ischemia cycles in rodents are among the most widely used models for experimental ulcers (Stadler et al. 2004; Huggenberger and Detmar 2011; Masson-Meyers et al. 2020). In many cases, these models are used without distinction to test the efficacy of different treatments, in spite of the fact that open and closed lesions have completely different underlying pathologies and cellular mechanisms. The aim of this study is to offer a comparative analysis of the cellular and molecular mechanisms underlying experimental wound healing, considering two main variables when determining the evolution of a chronic non-healing wounds. The first is related to a systemic disease, in this case diabetes, while the second is related to the mechanism of wound induction, comparing open (skin excision) and closed lesions (dermal hypoxia). For this purpose, we included diabetic (db/db) and non-diabetic (C57BL/6 J) mice in the study, in which both open (full-thickness skin excision, briefly indicated as “excision” in the text) and closed (hypoxia–ischemia repeated cycles, briefly indicated as “PrU” – pressure ulcer—in the text) skin lesions were induced. In addition to conventional morphological (histology and immunohistochemistry) and molecular analysis (single gene RT-PCR) focusing on well-known mechanisms and targets (re-epithelization, angiogenesis, and re-innervation), we also adopted a discovery strategy using mRNA arrays for about 240 genes encoding for proteins of the extracellular matrix, angiogenesis, and growth factors to identify novel potential molecular targets in the skin.

## Materials and methods

### Animals, lesions, and monitoring

All animal protocols described herein were carried out according to the European Community Council Directives (2010/63/EU), complied with the ARRIVE guidelines and the NIH Guide for the Care and Use of Laboratory Animals, and were approved by the Italian Ministry of Health (authorization no. 391/2017-PR). Eleven-week-old genetically diabetic male C57BL/KsJ-m + / + Leprdb (db/db) and C57BL/6 J mice were included in the study (Charles River Laboratories-Calco-Lecco), as detailed:

- Experiment 1, sacrifice at 50% closure:
-
  - db/db intact, *N* = 6
  - db/db full excision, *N* = 6
  - db/db pressure ulcer, *N* = 6
  - C57BL/6J intact, *N* = 6
  - C57BL/6J full excision, *N* = 6
  - C57BL/6J pressure ulcer, *N* = 6
- Experiment 2, sacrifice at closure:
-
  - db/db intact, *N* = 6
  - db/db full excision, *N* = 10
  - db/db pressure ulcer, *N* = 10
  - C57BL/6J intact, *N* = 10
  - C57BL/6J full excision, *N* = 10
  - C57BL/6J pressure ulcer, *N* = 10

In this last group, skin samples from 4 animals were used for morphology, and skin samples from 6 animals, for molecular biology. Db/db mice were randomly assigned to the groups in experiment 1 and 2 after blood glucose assay (acceptance criteria: glucose level ≥ 250 mg/dL); C57BL6 upon arrival.

The animals were housed with food pellets and water ad libitum, and a dark–light cycle of 12 h, and blood glucose was measured prior to the experiment (Contour XT, Bayer, Basel, Switzerland).

For lesion induction, the mice were deeply anesthetized (isoflurane 3% plus 2 l/min O_2_), shaved on the back, and the area thoroughly cleansed. For the skin excision model, a 6-mm circular full-thickness wound was created by dermal punch biopsy (BP-60F, Kai-Medical Industries Co. Ltd, Oyana, Seki City – Japan) on the midback of the mouse. PrUs were induced using two magnetic disks of 12 mm diameter (anisotropic ferrite) and a thickness of 5.0 mm, with an average weight of 2.4 g and 1000 G magnetic force (Algamagnetic, Italy). A skin fold was gently raised and placed between the two magnets. This procedure left a 1.0-cm skin bridge between the magnets creating about 50 mm Hg compressive pressure between the two plates, as has been documented to be necessary to cause local tissue ischemia (Stadler et al. 2004). Three ischemia–reperfusion (I/R) cycles were used, where a single I/R cycle consisted of a period of 12 h for the application of the magnets, followed by a rest period of 12 h without magnets. On day 3 following the final I/R cycle, surgical wound curettage was performed to remove the ischemic skin and eschar.

In both models, the wound area was covered with the semipermeable film dressing Tegaderm (Tegaderm Roll -3 M Health Care, St. Paul, MN, USA), and changed weekly until wound healing was complete. The animals were monitored daily for dressing integrity and infection, photographs were taken to monitor the wounds (Nikon C-Leds camera and X-Entry Alexasoft software), and the wound area was measured using Nis-Elements AR 3.2 software (Nikon Corporation, Tokyo). Since blinding procedure is not appliable for the ulcer types being clearly distinguishable, two independent operators were involved, and results are the mean of the relative measurements.

### Histology, immunohistochemistry, and image analysis

The samples collected for histology were biopsied (1 × 1 cm), fixed in paraformaldehyde 4% (w/v) and picric acid saturated aqueous solution in Sörensen buffer 0.1 M pH 7, embedded in paraffin, sectioned at 4 µm, and stained using hematoxylin and eosin (HE). The samples collected for immunohistochemistry were rapidly removed and fixed as above for 24 h, then washed for at least 48 h in 5% sucrose in 0.1 M phosphate buffer. After freezing in CO_2_, Sects. (14 µm thick) were cut using a cryostat (HM550 Microm, Bio-Optica). Sections were collected on gelatin-coated slides which were initially incubated in 0.1 M phosphate-buffered saline (PBS) at room temperature for 20 min, followed by overnight incubation at 4 °C in a humid atmosphere with the primary antibodies diluted in 0.3% Triton X-100/PBS (v/v). The following antisera were used in this study: PGP9.5 (Rabbit, ProteinTech, 1:150) and PECAM-1 (Goat, R&D systems, 1:150). After rinsing in PBS for 20 min, the sections were incubated at 37 °C for 30 min in a humid atmosphere with the secondary antisera conjugated with Rhodamine Red^™^-X-conjugated—affinity-pure Donkey anti-Rabbit IgG (Jackson Immunoresearch) or secondary DyLight 488 Donkey anti-Goat IgG (Thermo Fisher Scientific), diluted 1:100 or 1:200 in 0.3% Triton/PBS. The sections were then rinsed in PBS (as above) and mounted in glycerol containing 1,4-phenylendiamine (0.1 g/L). Six animals/groups were included in these experiments.

The measurement of repaired epidermal thickness was carried out on HE–stained sections. The mean value of five measurements/section and two sections per animal was used for the statistical analysis.

Immunofluorescence images were captured using a Nikon Eclipse E600 microscope equipped with a Q Imaging Retiga-2000RV digital CCD camera (Q Imaging, Surrey, BC, Canada) and a z-axis motorized stage. Analyses were performed using the Nis-Elements AR 3.2 software, by applying the same procedure to all images under comparison. Briefly, five z-stakes every 2 µm were collected from each image (300 × 500 µm), and the maximum intensity projection was used to calculate the immunoreactive area. For all morphological analyses, five images and two levels/animal sampled in the epidermal papillae at the center of the repaired ulcer were analyzed in each animal. All analyses were performed blindly. The immunoreactive area was calculated as area/fraction (percentage of PECAM-1 and PGP9.5 over 400 × 300 µm area).

For the figure preparation, immunofluorescence images were occasionally contrasted, and the same procedure was applied to the images under comparison.

### NGF and VEGF assay

Nerve growth factor (NGF) and vascular endothelial growth factor (VEGF) were dosed in plasma. Following blood collection, the EDTA-K2 Vacutainer tubes were centrifuged at 3000 × g for 10 min at 4 °C and the plasma aliquots stored at − 80 °C until use.

The assay was carried out using xMAP technology and a MAGPIX Luminex platform. The Human Adipokine Magnetic Bead Panel 2 high sensitivity kit (HADK2MAG-61 K, EMD Millipore Corporation, Billerica, MA, USA) was used to quantify mNGF, whereas the Mouse Cytokine/Chemokine Magnetic Bead Panel (MCYTOMAG-70 K, EMD Millipore Corporation, Billerica, MA, USA) was used to quantify mVEGF.

In both cases, the manufacturer’s protocol stipulated a first incubation with specific antibody conjugated beads (overnight at 4 °C), a second incubation with detection antibodies (1 h at RT), and a third with the streptavidin–phycoerythrin conjugated solution (30 min at RT), before reading on the MAGPIX instrument. Results were expressed as pg/ml.

mVEGF fluorescence values were interpolated on the standard curve provided by the kit (dynamic range 0.64–10.000 pg/ml) using xPONENT 4.2^®^ software and results expressed as pg/mL. mNGF fluorescence values were interpolated on a standard curve obtained by serial dilutions of mouse NGF protein (dynamic range 0.64–10.000 pg/ml) and the results expressed as pg/mL. Assay performance was evaluated using a standard curve correlation coefficient (*R*^2^ value > 0.98) and the quality controls included in the kits (pg/ml values within the range specified by the kit manufacturers). All samples were dosed in duplicate and a variability of CV < 20% was considered acceptable.

### Western blot

Western blot was used to quantify P-Akt/Akt protein expression in intact skin. Following homogenization (ratio 1:8 = mg:µL) in RIPA buffer and protease inhibitor (1 × cocktail inhibitor Sigma, 1 mM PMSF, 10 mM sodium fluoride, 1 mM sodium orthovanadate), tissues were centrifuged at 12,000 g for 20 min at 4 °C. Supernatants were collected, boiled at 100 °C for 5 min with a denaturating solution (Leammli/β-mercaptoethanol), and used to perform western blot analysis. A standard colorimetric method based on the Lowry assay (DC Protein Assay, Bio-Rad) was used to estimate total protein concentration. A total of 25 µg of total protein and the chemiluminescent marker (Precision Plus Protein Standards, Bio-Rad) were loaded on to the gels.

A total of 4–20% Mini-PROTEAN TGX Stain-Free Gels (Bio-Rad) were used to resolve the proteins, Amersham Protran 0.45 µm Nitrocellulose Blotting Membrane (Bio-Rad) was used to transfer, and Tris Buffer Saline solution containing 1% Tween20 (TBST) and 2.5% BSA solution was used to block the non-specific interaction sites before immunoblotting.

Phospho-Akt (P-Akt), pan-Akt (Akt, rabbit, Cell Signaling -Leiden, The Netherlands- 1:1000), and β-actin (Santa Cruz Biotechnology -Dallas, Texas- 1:150) were used as primary antibodies, whereas anti-rabbit (Goat Anti-Rabbit IgG (H + L)-HRP Conjugate Biorad, 1:5000) and anti-mouse (Goat Anti-Mouse IgG (H + L)-HRP Conjugate Biorad, 1:5000) were used as secondary antibodies. The first incubation was performed overnight at 4 °C, and the second for 1 h at room temperature. TBST was used to wash the membranes 3 times after each incubation.

Clarity Western ECL Substrate (Bio-Rad) for 5 min at RT in darkness and BioRad Chemi DOC MP imaging systems were used to detect the immunoreactive signal. Densitometry analysis to quantify signal intensity was performed using ImageJ software (Fiji, version 1.53c), and ratios between pan-Akt on β-actin and P-Akt on pan-Akt were used to compare groups.

### mRNA extraction

The core of the skin lesion (6 mm diameter) was used for total RNA isolation using the RNeasy Mini kit (Qiagen, Milan, Italy) following the manufacturer’s instructions. Total RNA was eluted in RNase Free Water, and the concentration was estimated through absorbance reading (Nanodrop 2000 spectrophotometer, Thermo Scientific). First strand cDNAs were obtained using the iScript^™^ cDNA Synthesis Kit (BioRad), incubating at 42 °C for 30 min. An RNA sample with no reverse transcriptase enzyme in the reaction mix was processed as a no-reverse transcription control sample.

### RT-PCR

Semi-quantitative real-time PCR was performed using the CFX96 real-time PCR system (BioRad, CA, USA). The reactions were performed in a final volume of 20 µL consisting of 1 × SYBR Green qPCR master mix (BioRad) and 0.4 µM forward and reverse primers. To avoid possible contamination of genomic DNA in isolated RNA, the sample with the non-reverse transcriptase enzyme was processed in parallel with the others and tested by real-time PCR for every pair of primers used. All primers used were designed using Primer Blast software (NCBI, MD, USA) and synthesized by IDT (Coralville, IA, USA). The following primer sequences were used: *Gapdh*, as housekeeping gene (FW: 5′-GGCAAGTTCAATGGCACAGTCAAG-3′; REV: 5′ACATACTCAGCACCAGCATCACC-3′); *Vegfa* (FW: 5′-AAGAGAAGGAAGAGGAGAG-3′; REV: 5′-ACCCAAGAGAGCAGAAAG-3′); *Flt1* (FW: 5′-CGTGCAAGGAACCTCAGACA-3′; 5′-ATCATAGGGCAGCCGTTCAC-3′); *Kdr* (FW: 5′-ATGTCCTTGGCTGTGCAAGA-3′; REV: 3′-CCTTCATTGGCCCGCTTAAC); *Hif1a* (FW: 5’-CACAGAAATGGCCCAGTGAGA-3’; REV: 5’-ATGAATATGGCCCGTGCAGT-3’); *Ngf* (FW: 5’-ACCTCTTCGGACACTCTG -3’; REV: 5’- CGTGGCTGTGGTCTTATCTC-3’); *TrkA* (FW: 5’- TGCCTTCCGTTTCACCCCTCG-3’; REV: 5’- CCCTTCCTGCTCCCAACGCT-3’); and *p75*^*NTR*^ (FW: 5’- AGTGGCATCTCTGTGGAC-3’; REV: 5’- CTACCTCCTCACGCTTGG-3’).

To check for possible mix contaminations, a duplicate of the no-template control not containing cDNA was added to the plate for each primer mix.

The housekeeping gene expression was used to normalize the amount of reverse-transcribed RNA used for PCR. The thermal profiles of the PCR reactions consisted first of a denaturation step (95 °C, 2 min) and 40 cycles of amplification (95 °C for 15 s and 60 °C for 60 s). At the end of the amplification cycles, the melting curve of the amplified products was performed according to the following temperature/time scheme: heating from 55 to 95 °C with a temperature increase of 0.5 °C/s.

Primer efficiency values for all primers were 95–102%. The 2 ^(−ΔΔCT)^ method was used to calculate gene expression.

### PCR arrays

The exploratory data-driven strategy on the wound healing study of the two different skin lesions in C57BL/6 J and diabetic mice was investigated using a pathway-focused gene expression analysis using the RT2 Profiler PCR Arrays (Qiagen), focusing on the main molecular pathways in the healing process, i.e., angiogenesis, extracellular matrix (ECM), and growth factors (GFs), in skin sampled at 50% of the repair process. Samples were collected from both intact animals and from the PrU and excision groups at the core of the lesion (6 mm diameter), and RNA was extracted from all animals (6 animals per group), quantified (Nanodrop 2000 spectrophotometer), and pooled (100 ng per animal). A total of 600 ng of RNA per group was therefore used for the reverse transcription.

The pooled RNAs were retrotranscribed using the RT2 First Strand Synthesis Kit (Qiagen) according to the manufacturer’s instructions. Each pooled group was tested using a single PCR array, using the CFX96 real time PCR instrument (BioRad).

The Qiagen mouse angiogenesis (PAMM-024Z), extracellular matrix and adhesion protein (PAMM-013Z), and neurotrophins and growth factors (PAMM-031Z) were used to profile the expression of more than 250 key genes involved in these pathways (see Supplementary materials, Table S1, for the full list).

The dedicated Qiagen online data analysis software (GeneGlobe) for relative quantification of the gene expression was used to perform the analysis and generate the graphs. Data were normalized on a housekeeping gene automatically selected by the software among the whole gene list (Thrombospondin1, *Thbs1*).

### Bioinformatic analysis

The differentially expressed genes identified from the analyzed groups were plotted in the STRING network analysis software (v.10; http://string-db.org/) to analyze the functional interactions between the biological functions of the encoded proteins.

To compare the intact groups and the PrU between the two genotypes, the Gene Codis software (v.4.0; https://genecodis.genyo.es/) was also used for the pathway enrichment analysis, using different algorithms (KEGG, Panther, Reactome).

### Statistical analysis

The data is expressed as mean ± SEM. The number of animals included in each experiment is indicated in the figure legends. Statistical comparison of the rate of skin ulcer healing over time between genotypes was performed by two-way ANOVA. Student’s *t*-test or one-way ANOVA was used to analyze the data (GraphPad Software, San Diego, CA, USA), and results were considered significant when the probability of their occurrence as a result of chance alone was less than 5% (*p* < 0.05).

## Results

### Wound evolution in diabetic and non-diabetic mice

Blood glucose was measured in the db/db mice, and only mice showed a blood glucose level of ≥ 250 mg/dL, thus were included in the study. The animals underwent body weight monitoring, and the diabetic mice were already obese at the beginning of the experiment as expected (db/db: g 43.13 ± 1.5; C57BL/6 J: g 24.5 ± 0.5; Student’s *t*-test, *p* < 0.0001; Table 1).

**Table 1:** Body weight prior to lesion induction (intact) and at sacrifice in C57BL/6 J and db/db mice in experiment 2

Genotype	Intact, day 0	Intact	Full-excision	Pressure
C57BL/6 J	23.38 ± 0.40 g	23.75 ± 0.47 g	24.50 ± 0.50 g	24.80 ± 0.37 g
db/db	40.63 ± 0.96 g	43.13 ± 1.55 g	36.50 ± 1.30 g *	35.07 ± 1.36 g *

Statistical analysis: one-way ANOVA and Dunnett’s multiple comparison test (intact, sacrifice, as control group)

^*^p < 0.05

**Fig. 1:**
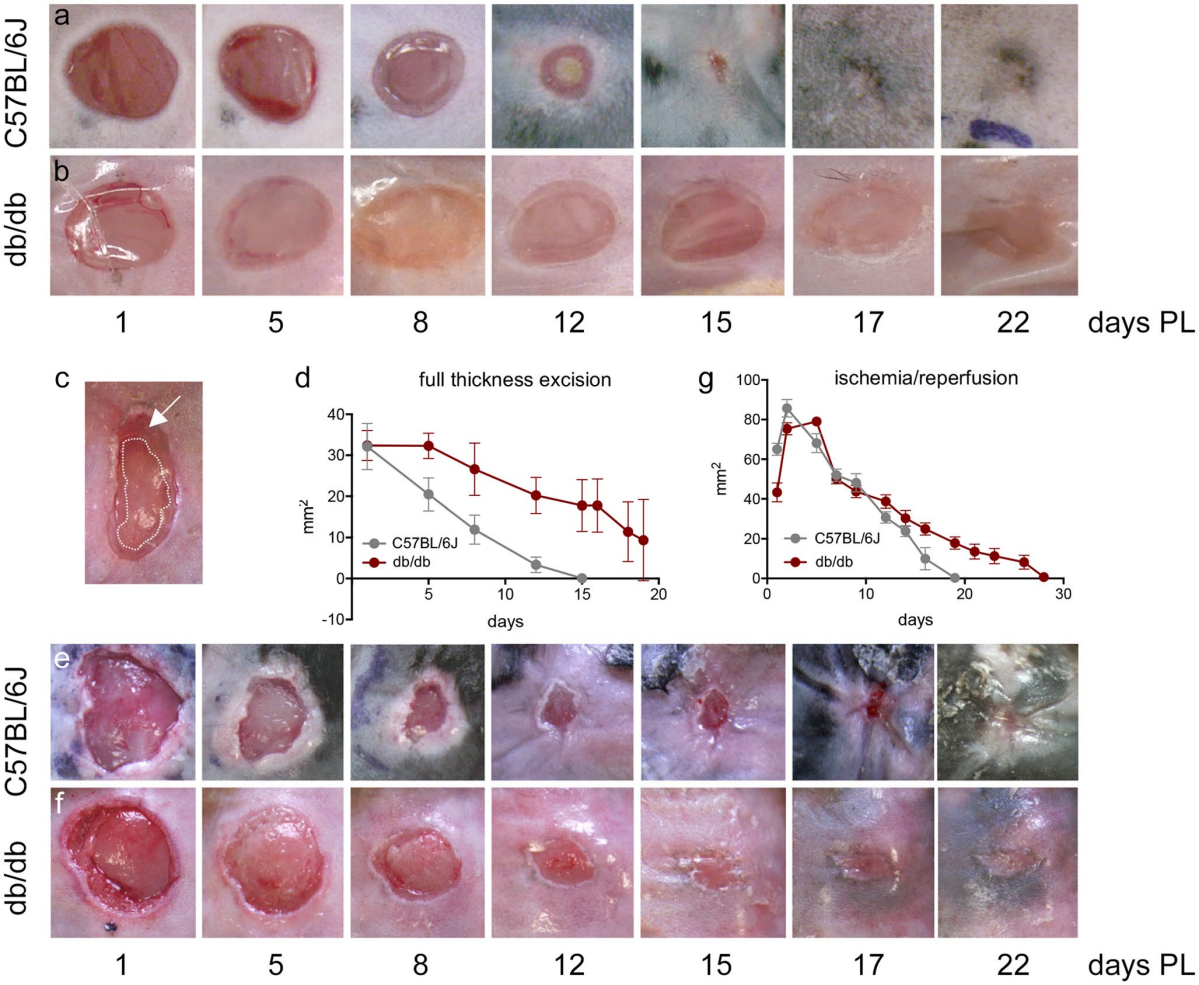
Lesion monitoring in diabetic and non-diabetic mice. Representative photos of the full-thickness skin excision ulcers at different post-lesion times in C57BL/6 J **a** and db/db **b** mice. Sampling strategy for the re-epithelization analysis. The arrow indicates the area covered by a thin layer of epithelial cells, while the dotted line indicates the border of the non-re-epithelized area, used for the analysis **c**. Ulcer areas in the experimental groups over the observational days. *N* = 10 ulcers/groups were included in this experiment. Statistical analysis: two-way ANOVA, *p* < 0.0001 for time and genotype **b**. Representative photos of the pressure ulcers at different post-lesion times in C57BL/6 J **e** and db/db **f** mice. Ulcer areas in the experimental groups over the observational days. *N* = 10 ulcers/groups were included in this experiment. Statistical analysis: two-way ANOVA, *p* < 0.0001 for time and genotype **g**

Wound repair was monitored photographically from day 0 (day of surgery) until sacrifice and evaluated by measuring the area defined by the re-epithelialization edges at the different times (see Fig. 1c for sampling strategy). *N* = 10 ulcer/genotype/lesion types were monitored in this experiment. Photographs of the full-thickness excision wounds in representative C56BL6 and db/db mice are shown in Fig. 1a, b, and the time course of the reduction in lesion area in the two groups is given in Fig. 1d. A significant delay in db/db mice was observed, with healing complete at 16 days in all C57BL/6 J mice, whereas the process was still incomplete at 20 days post-lesion in some db/db mice (two-way ANOVA, *p* < 0.0001).

Photographs of the ischemia/reperfusion pressure wounds in representative C56BL6 and db/db mice are shown in Fig. 1e, f, and the time course of area reduction in the two groups is given in Fig. 1g. We observed a significant wound repair delay in db/db mice in this model also, with healing complete at 19 days in all C57BL/6 J mice, while the process was not complete until 28 days post-lesion in db/db mice (two-way ANOVA, *p* < 0.0001).

### Angiogenesis and re-innervation in excision wound and pressure ulcer in diabetic and non-diabetic mice

**Fig. 2:**
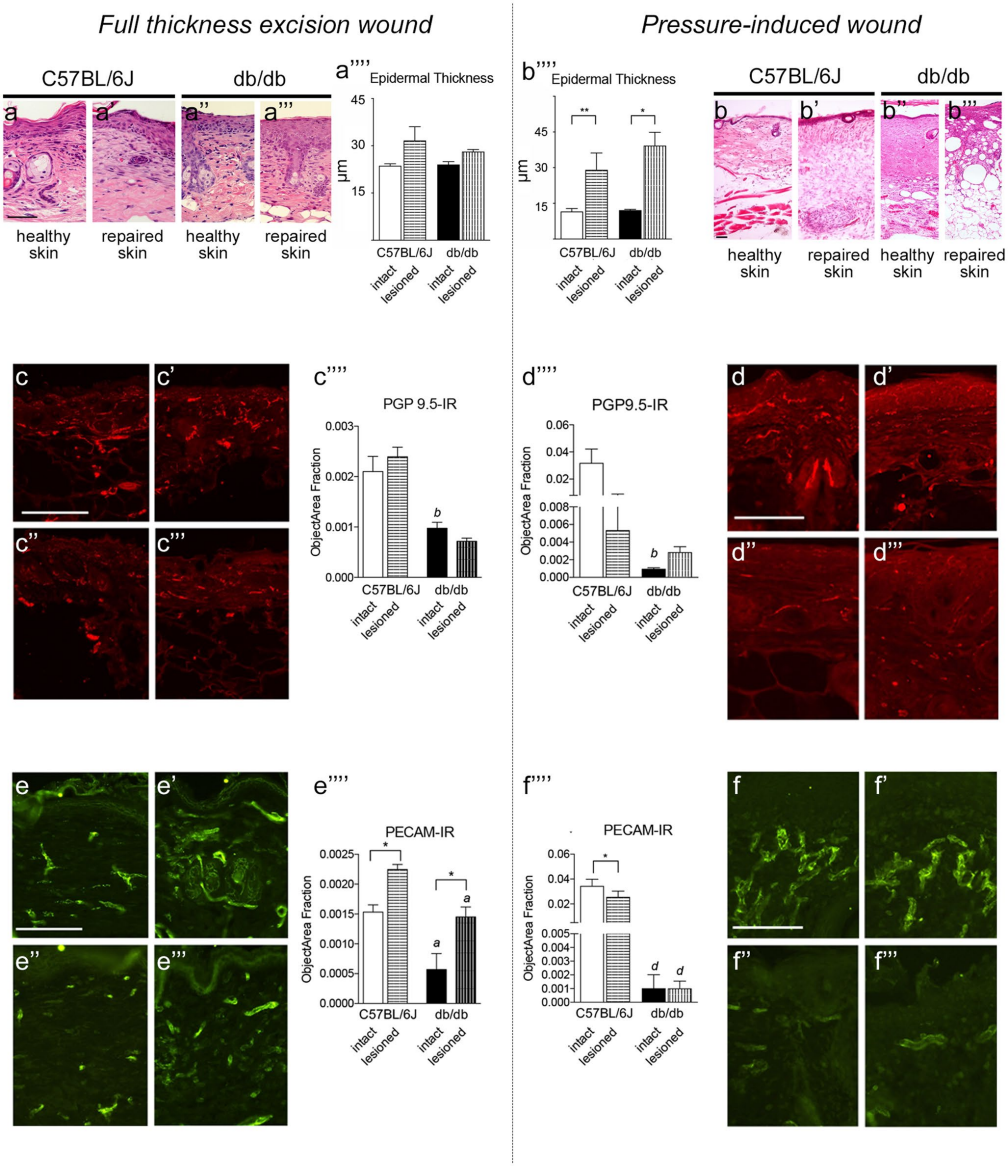
Histological and immunohistochemical analysis of the repaired skin in the full-thickness excision and pressure model. *N* = 5 animals/groups were included in this experiment. Re-epithelization in C57BL/6 J and db/db mice in full thickness excision wound **a**–**a’’’’** and pressure-induced wound **b**–**b’’’’**. In each model, representative micrographs of H&E staining of the intact and repaired skin and the relative morphometric measure are shown. Re-innervation in C57BL/6 J and db/db mice in full thickness excision wound **c**–**c’’’’** and pressure-induced wound **d**–**d’’’’**. In each model, representative micrographs of PGP9.5-IR fibers in the intact and repaired skin and the relative morphometric measure are shown. Capillary density in C57BL/6 J and db/db mice in full thickness excision wound (**e**–**e’’’’**) and pressure-induced wound (**f**–**f’’’’**). In each model, representative micrographs of PECAM-IR fibers in the intact and repaired skin and the relative morphometric measure are shown. Data are presented as mean ± SEM. Statistical analysis: Student’s *t*-test: intact vs lesioned for each genotype, *p < 0.05; **p < 0.01; C57BL/6 J vs db/db mice, *a p* < 0.05, *b p* < 0.01, *c p* < 0.001. Bars: 50 µm

We first analyzed the histological, immunohistochemical, and molecular parameters related to well-known mechanisms which determine wound evolution, i.e., re-epithelization, re-innervation, and angiogenesis at wound closure. Results of the morphological and morphometric analysis, re-innervation, and angiogenesis at wound closure in the excision and pressure models in C57BL/6 J and db/db mice are shown in Fig. 2.

The re-epithelization was analyzed by measuring the epidermal thickness at the equator of the lesioned areas. Representative images of HE staining in C57BL/6 J and db/db mice in healthy and repaired skin are shown in Fig. 2a–a’’’’ (full excision wound) and b–b’’’’ (pressure ulcer), where the graph shows the epidermal thickness. No differences were observed between C57BL/6 J and db/db mice, and the pressure lesion induced a hypertrophic reaction at wound closure in both genotypes.

The nerve endings in the skin were analyzed by PGP9.5-IR, the most popular marker for epidermal nerve ending quantification. Representative immunofluorescence images in C57BL/6 J and db/db mice in healthy and repaired skin are shown in Fig. 2c–c’’’’ (excision wound) and d–d’’’’ (pressure ulcer), where the PGP9.5-IR percentage area in the epidermal papillae of the repaired area is also shown in the graph. Skin sensory innervation was reduced in db/db compared to C57BL/6 J. In repaired skin at the investigated time point, no differences were observed compared to the corresponding genotype in both lesion models.

The skin capillary plexuses were analyzed by PECAM-IR (platelet endothelial cell adhesion molecule—PECAM-1—also known as cluster of differentiation 31-CD31). Representative immunofluorescence images in C57BL/6 J and db/db mice in healthy and repaired skin are shown in Fig. 2e–e’’’’ (excision wound) and f–f’’’’ (pressure ulcer), where the PECAM-IR percentage area in the derma of the repaired area is also shown. A dramatic decline in capillary density was observed in the healthy skin of db/db compared to C57BL/6 J. In addition, angiogenesis was stimulated in the full-excision wound, which did not occur in the PrU of either genotype.

**Fig. 3:**
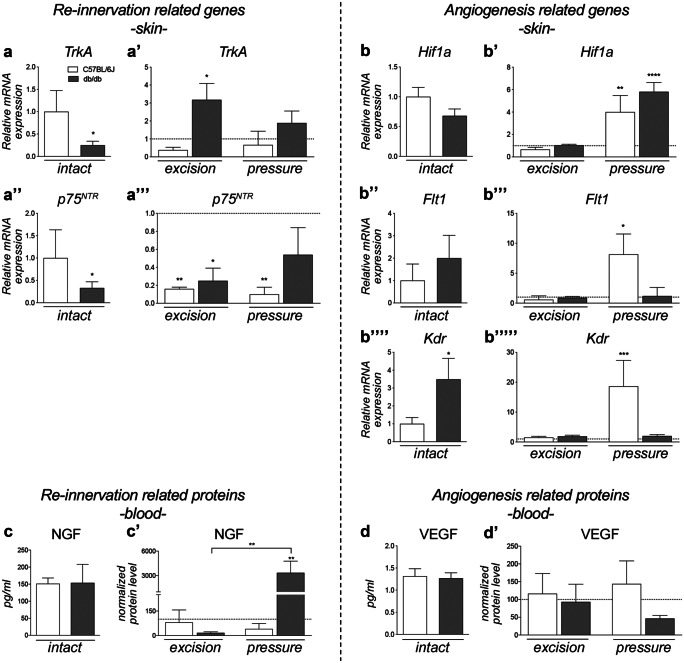
Gene expression and protein quantification of re-innervation and angiogenesis-related molecules. Graphs show the relative expression analysis of *TrkA* **a**, **a’** and *p75*^*NTR*^ **a’’**, **a’’’** or *Hif1a ***b**, **b’**, *Flt1* **b’’**, **b’’’**, and *Kdr* **b’’’’**, **b’’’’’** genes normalized on C57BL/6 J for the intact group analysis **a**, **a’’**, **b**, **b’’**, **b’’’’**, or on genotype-specific intact groups (horizontal dotted line) for the ulcer analysis **a’**, **a’’’**, **b’**, **b’’’**. Graphs show the quantification of the Ngf and Vegf proteins in the plasma of C57BL/6 J and db/db animals, in intact conditions or ulcers **c-d’**. In the comparison between the intact groups **c**, **d**, the absolute concentration of the protein is shown, while in the comparison between ulcers **c’**, **d’**, the quantification is normalized on the genotype-specific intact group (horizontal dotted line). Data are presented as mean ± SEM. *N* = 6 animals/groups were included in this experiment. Statistical analysis: C57BL/6 J vs db/db intact, Student’s *t*-test. Ulcer comparison, one-way ANOVA followed by Dunnett’s post-test versus the genotype-specific intact group **a**–**b’’’’’** or Tukey’s post-test **c-d’**. Asterisks represent the differences versus the control group or between two groups indicated by an horizontal line (*p < 0.05; **p < 0.01; ****p < 0.0001)

Considering that NGF is the growth factor responsible for the development and maintenance of sensory skin innervation in adulthood (Indo 2010), we performed mRNA expression analysis of the *Ngf*, NGF high- (*TrkA*) and low- (*p75*^*NTR*^) affinity receptors at 50% of wound closure (Fig. 3a, a’’’). The *Ngf* mRNA expression level was undetectable in the skin in the investigated experimental conditions and at the investigated time-points. We found that both the *TrkA* and *p75*^*NTR*^ mRNA expression levels were much lower in the intact skin of db/db compared to C57BL/6 J mice (Student’s *t*-test; *TrkA*, *p* = 0.0219; *p75*^*NTR*^, *p* = 0.0140). Both lesions induced an upregulation of *TrkA* in db/db mice (one-way ANOVA, *TrkA* F(2,13) = 4.577, *p* = 0.0313), which was statistically significant in the excision model (Dunnett’s post-test, *p* = 0.0183), while no significant changes in the C57BL/6 J mice were observed. The expression of *p75*^*NTR*^ was modified by the lesions in both C57BL/6 J (one-way ANOVA, F(2,13) = 14.60, *p* = 0.0005), in excision (Dunnett’s post-test, *p* = 0.0014), and PrU (*p* = 0.005). In dbdb mice, *p75*^*NTR*^ expression was also modified (one-way ANOVA, F(2,13) = 4.379, *p* = 0.0352) but only in excision wound (Dunnett’s post-test, *p* = 0.0202).

We then investigated the main molecular players in angiogenesis by mRNA expression analysis of *Hif1a*, the main oxygen sensor in cells (Darby and Hewitson 2016), *Vegf*, and related receptors in the skin as HIF1α-dependent angiogenic factors, and the results are shown in Fig. 3b–b’’’’’. We found that *Hif1a* mRNA tends to be reduced in db/db compared to C57BL/6 J; *Vegfa* mRNA was detected at low levels (Ct > 30) in both genotypes with no differences between groups (*data not shown*), and the VEGF receptor was upregulated in db/db intact skin, being significant for *Kdr* (Student’s *t*-test, *p* = 0.0369). *Hif1a* mRNA was strongly upregulated in the closed (PrU induced by ischemic/reperfusion cycles) but not in the open (excision) wound in both C57BL/6 J (one-way ANOVA, F(2,14) = 14.38, *p* = 0.0004; Dunnett’s post-test, *p* = 0.0025) and db/db (one-way ANOVA, F(2,14) = 58.04, *p* < 0.0001; Dunnett’s post-test, *p* < 0.0001) skin. VEGF receptors were upregulated by skin lesions only in C57BL/6 J mice (one-way ANOVA; *Flt1*, F(2,14) = 5.160, *p* = 0.0210; *Kdr*, F(2,14) = 18.42, *p* = 0.0001) in PrU (Dunnett’s post-test; *Flt1*, *p* = 0.049; *Kdr*, *p* = 0.0001).

Given that NGF and VEGF are not produced locally in lesioned skin, we also measured the blood levels of these growth factors. While no differences were observed according to genotype, the PrU induced a dramatic increase in NGF in db/db but not in C57BL/6 J mice (one-way ANOVA, F(2,13) = 8.617, *p* = 0.0041; Tukey’s post-test; pressure vs intact, *p* = 0.0075; excision vs pressure, *p* = 0.0063) (Fig. 3c, c’).

Circulating blood levels of VEGF were similar in C57BL/6 J and db/db mice, and no significant changes were observed in either lesion type (Fig. 3d, d’).

### Gene pathway analysis in excision wounds and pressure ulcers in diabetic and non-diabetic mice

Following characterization of the two wound models via well-known pathways involved in ulcer onset and response, a data-driven approach was used to investigate possible mechanisms differentiating wound repair in skin excision and PrUs in C57BL/BJ and db/db animals, respectively. The analysis was carried out at 50% of wound repair, when inflammation and the proliferative phase are active. Three different PCR arrays were used, analyzing gene expression with 252 total inputs for ECM, angiogenesis, and growth factors. The fold change for each input gene was calculated using the QIAGEN data analysis software, pooling the entire dataset, and the results of the expression analysis are shown in Supplementary Table S1.

**Fig. 4:**
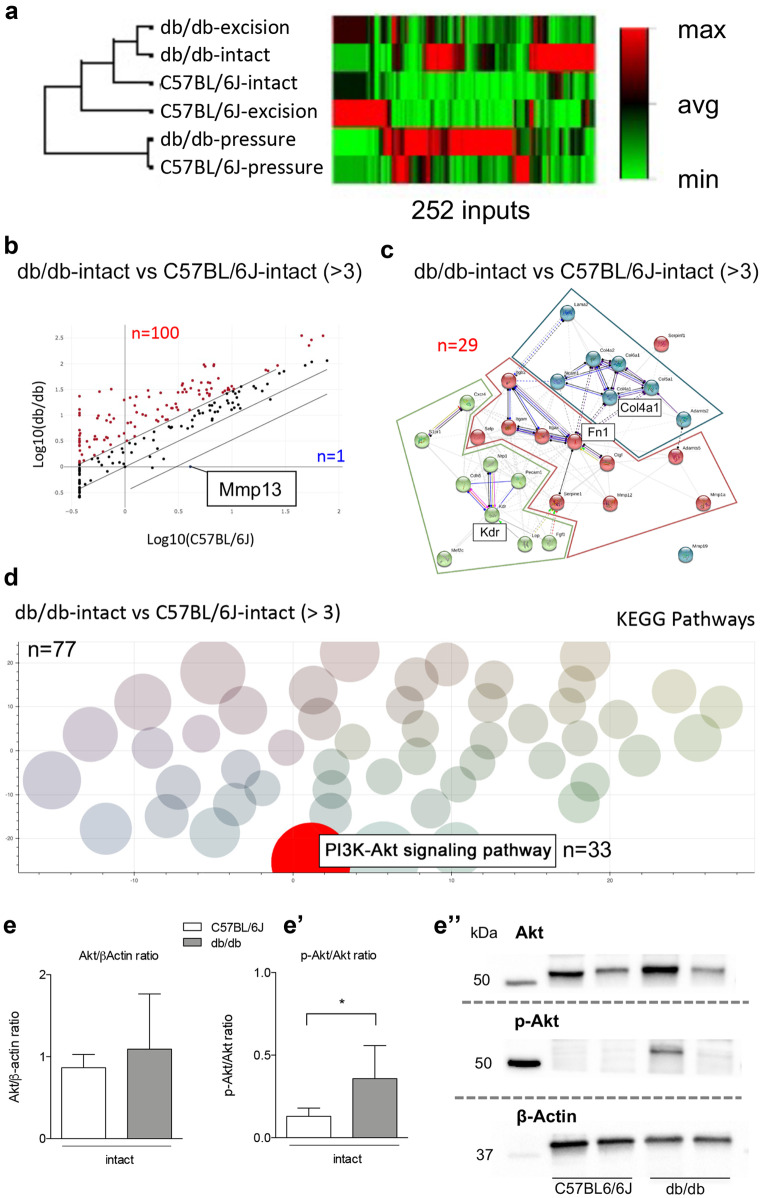
PCR arrays and western blot analysis: C57BL/6 J vs db/db intact skin. Cluster analysis of the 252 inputs obtained from the three PCR arrays plates for growth factors, extracellular matrix and related molecules, and neurotrophins **a**. Scatter plot representation of the gene expression fold change in db/db vs C57BL/6 J intact skin, using a fold change of 3 as cutoff value for significance gene expression variation **b**. STRING software–based protein interaction network analysis of proteins encoded by genes showing a fold of change higher than 10 in db/db vs C57BL/6 J intact skin **c**. Gene Codis 4.0 software pathway enrichment analysis based on the KEGG database, using the entire group of 101 genes differentially expressed in db/db vs C57BL/6 J intact skin (fold of change > 3). The KEGG database recognized 77 of the 101 input genes **d**. Graphs show the quantification by western blot of Akt **e** and p-Akt **e’**, expressed as a ratio on b-Actin and Akt (total), respectively, in C57BL/6 J and db/db intact skin. Representative images of quantified proteins for each group are shown in the figure **e’’**. PCR array experiments were performed on pooled RNAs (*N* = 6 animal/group). Statistical analysis: Student’s *t*-test. Asterisks represent the differences between db/db and C57BL/6 J (*p < 0.05)

We first performed a cluster analysis on ΔCq (Fig. 4a) based on the expression pattern of all input genes. The heat map representation shows that db/db and C57BL/6 J intact tissues clusterize with their genotype-matching skin excision model. On the contrary, the gene expression pattern in the PrU model clusterizes with both genotypes independently of both skin excision and intact tissue, suggesting that the strong gene expression regulation caused by this lesion type prevails over the genotype differences.

To better understand the changes in gene expression due to genotype, we analyzed the differential basal gene expression in C57BL/6 J and db/db intact animals. Three different approaches were used: (i) general differential gene expression (fold change ≥ 3, Fig. 4b, supplementary Table S2), (ii) protein–protein interaction analysis of proteins encoded by high-differential gene expression (fold change ≥ 10, Fig. 4c), and (iii) pathway enrichment analysis (Fig. 4d). Of the 100 genes upregulated in db/db skin, 29 were overexpressed at high level, showing a fold of change more than 10 times higher than C57BL/6 J (Fig. 4c). The STRING software analysis revealed three main clusters produced by the connection of the proteins encoded by the differentially expressed genes through a molecular interaction network, with *Kdr*, *Fn1*, and *Col4a1* as the center molecules of the different clusters. Using the entire dysregulated gene dataset (fold of change ≥ 3; *n* = 101) for pathway enrichment analysis as input in the Gene Codis software, the KEGG algorithm recognized and clusterized 77 genes, finding the PI3K-Akt signaling pathway to be the major dysregulated network in the db/db compared to the C57BL/6 J animals (Fig. 4d). This result was validated by WB analysis of the Akt protein in the skin (Fig. 4e–e’’), which revealed an altered P-Akt/panAk ratio in db/db mice.

**Fig. 5:**
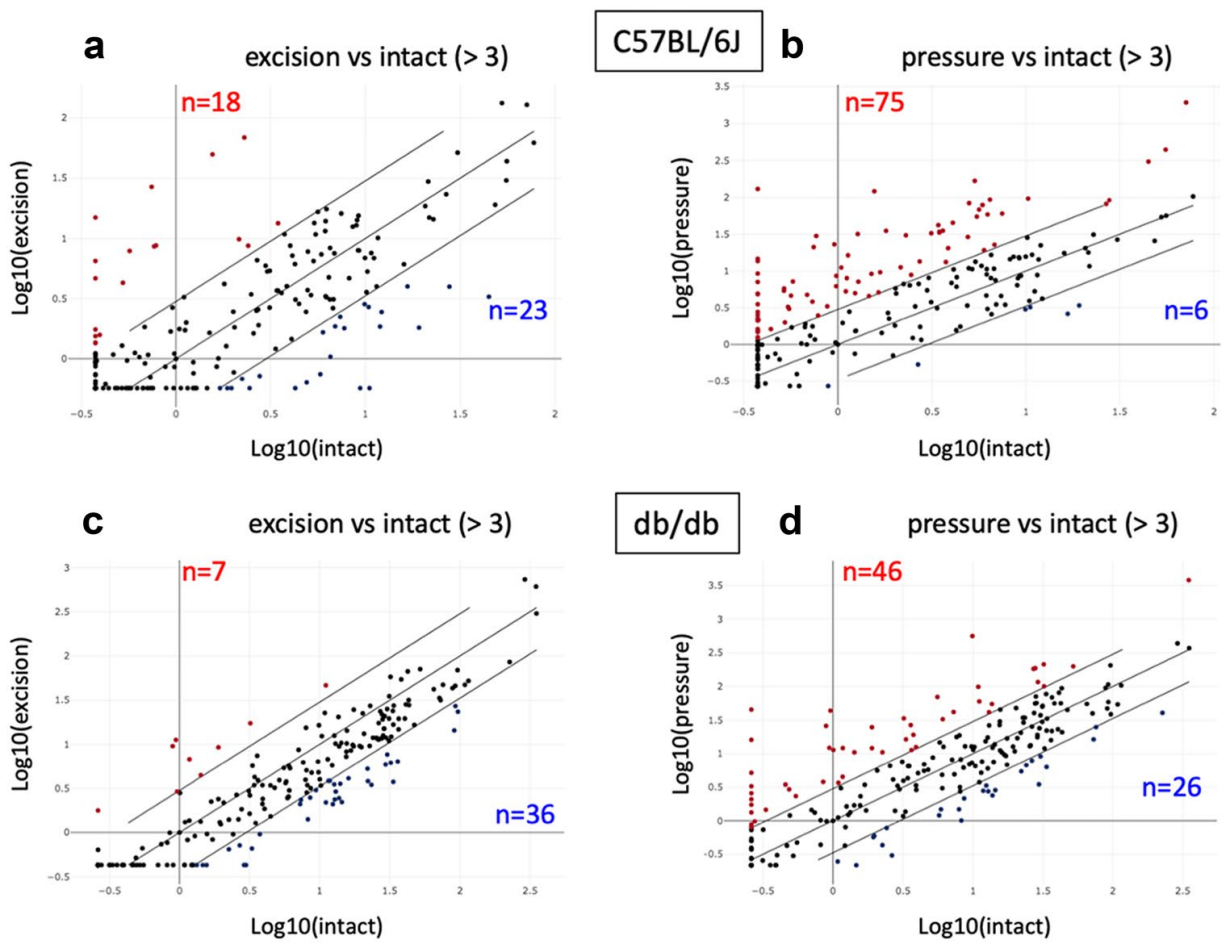
PCR array analysis: skin excision vs pressure ulcer in C57BL/6 J and db/db mice. Scatter plot representation of gene expression fold change in C57BL/6 J skin excision **a** or pressure ulcer **b**, and db/db skin excision **c** and pressure ulcer **d**, vs the genotype-specific intact skin, using a fold change of 3 as a cutoff value for gene expression variation significance. PCR array experiments were performed on pooled RNAs (*N* = 6 animal/group)

Following analysis of the basal gene expression in intact skin in the two genotypes, we compared the skin excision and PrU models in both C57BL/6 J and db/db mice. In C57BL/6 J animals, the skin excision produced an upregulation of 18 and a down-regulation of 23 genes (Fig. 5a), while PrU produced an upregulation of 75 genes and a downregulation of 6 genes (Fig. 5b). Skin excision produced a mostly downregulated gene expression, with 36 downregulated and 7 upregulated genes (Fig. 5c). As in the C57BL/6 J strain, PrU resulted in a higher number of regulated genes (46 upregulated and 26 downregulated) compared to excision (Fig. 5d) in db/db animals also, and the list of regulated genes is shown in the supplementary materials (Table S2).

**Fig. 6:**
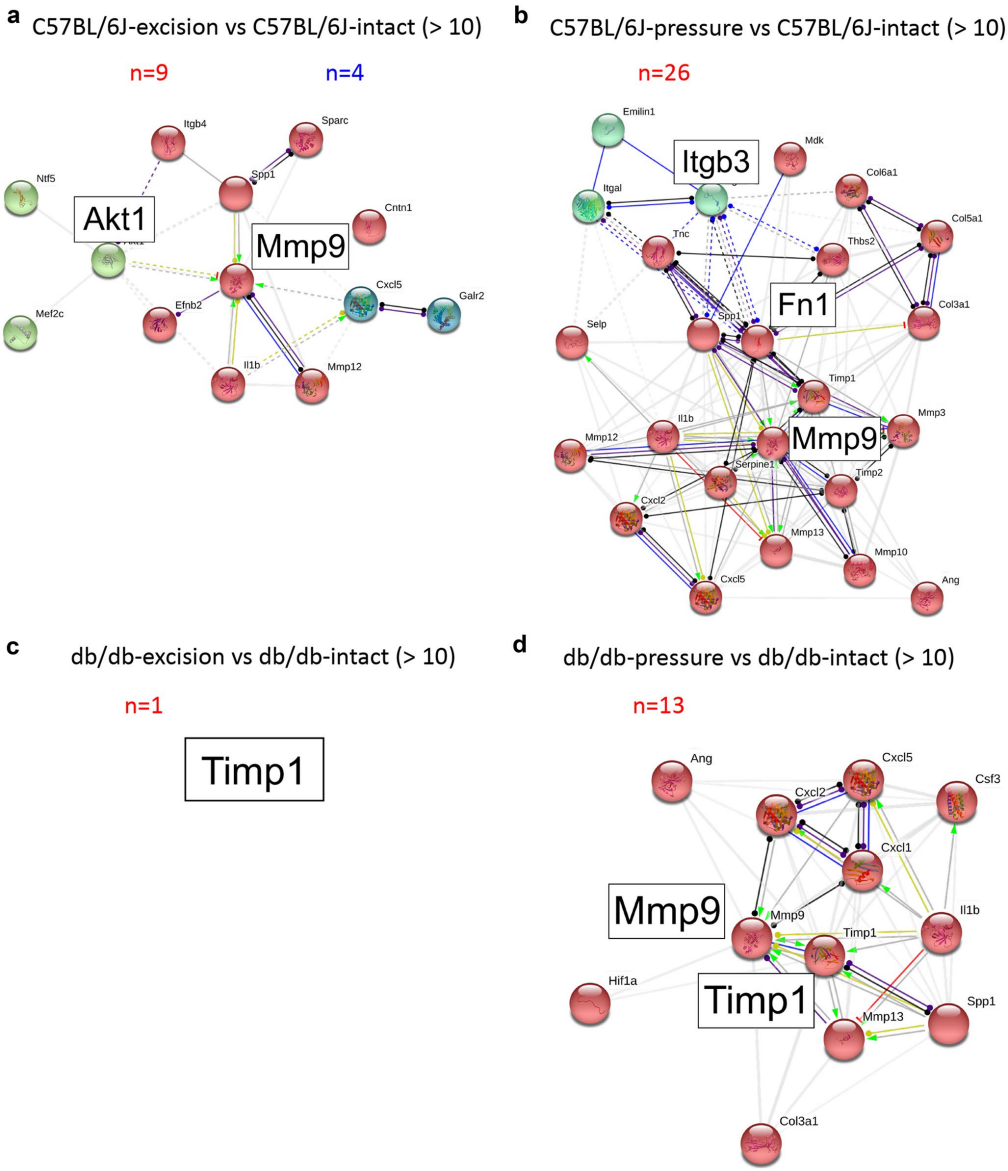
Protein–protein interaction analysis: skin excision vs pressure ulcer in C57BL/6 J and db/db mice. STRING software–based protein interaction network analysis of proteins encoded by genes showing a fold of change higher than 10 in C57BL/6 J skin excision **a** or pressure ulcer **b**, and db/db skin excision **c** and pressure ulcer **d**, vs the genotype-specific intact skin. PCR array experiments were performed on pooled RNAs (*N* = 6 animal/group)

The cluster analysis was performed to identify the “nodes” of the protein–protein interaction network activated in the different experimental conditions. For the excision model using stringent criteria (fold of changes ≥ 10), Akt1 and Mmp9 were the main network nodes identified in C57BL/6 J mice (Fig. 6a), while Timp1 was the only network node in db/db mice (Fig. 6c). As already mentioned, PrU induced a much more stronger gene expression regulation in both genotypes. Itgb3, Fn1, and Mmp9 were at the center of the protein functional network of PrU in C57BL/6 J mice (Fig. 6b), while Mmp9 and Timp1 were at the center of the network in db/db mice (Fig. 6d).

**Fig. 7:**
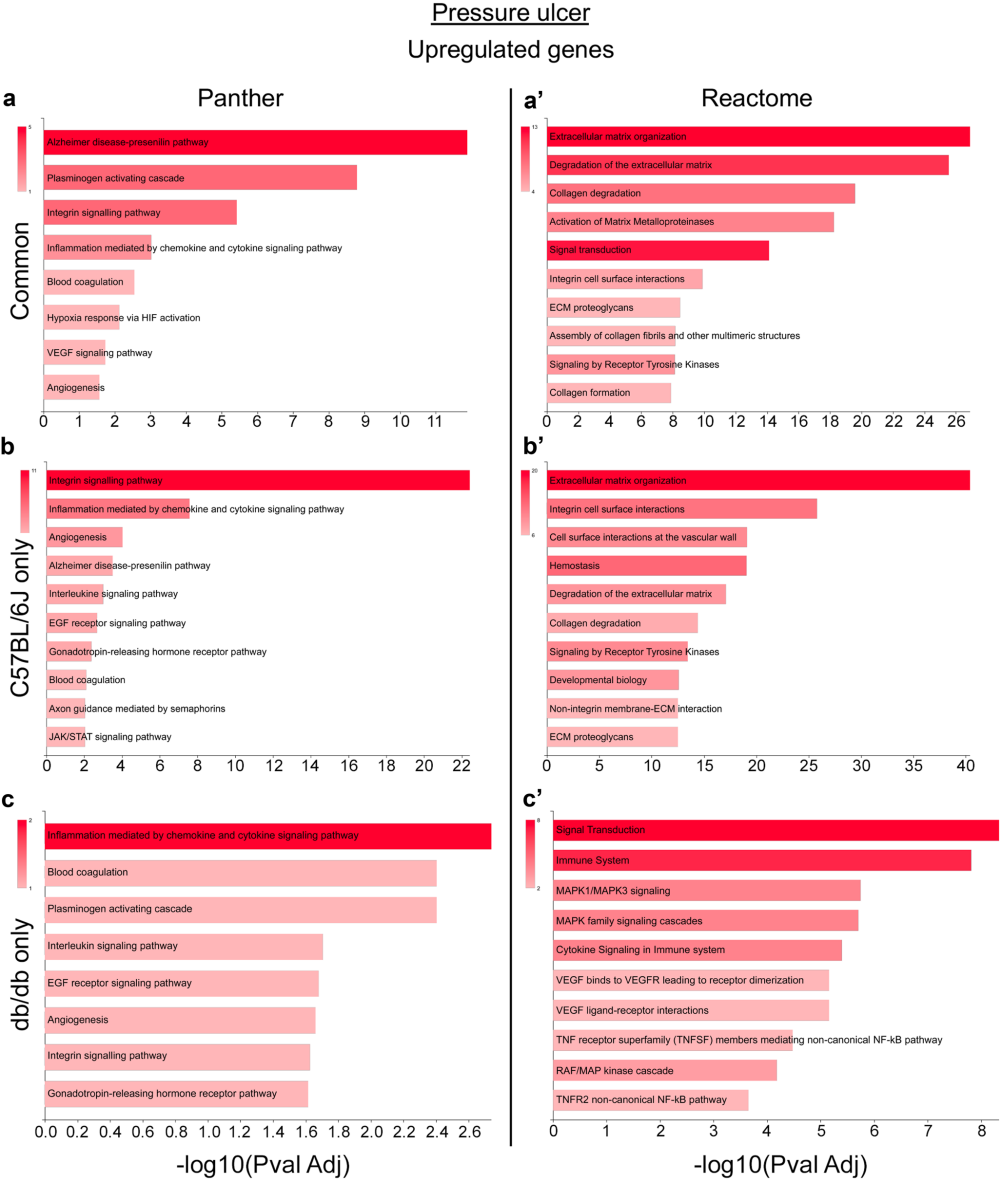
Pathway enrichment analysis of common and differentially upregulated genes by pressure ulcer in C57BL/6 J and db/db mice. Pathway enrichment analysis performed by GeneCodis 4.0 using Panther and Reactome databases for common genes upregulated by pressure ulcers in C57BL/6 J and db/db using Panther **a** or Reactome **a’** algorithm, and genes upregulated only in C57BL/6 J, using Panther **b** or Reactome **b’** algorithm, or only in db/db using Panther **c** or Reactome **c’** algorithm. Only significant pathways are shown (*p* < 0.05). Color gradient represents the gene number in each identified pathway, while the -log (adjusted *p* value) is shown on the horizontal axis. PCR array experiments were performed on pooled RNAs (*N* = 6 animal/group)

The PrU evoked a higher gene expression regulation in both genotypes, with mainly upregulated genes. To investigate the pathway activated by this upregulation in the two genotypes, we compared the upregulated genes in the C57BL/6 J and db/db animals of the PrU group. We initially divided the upregulated and downregulated genes in three different groups: those shared by the two genotypes, those upregulated only in C57BL/6 J, and those upregulated only in db/db (Supplementary Table S3). We then attempted to perform a pathway enrichment analysis of the upregulated genes using two different databases (Panther and Reactome) on the three groups (the full list of the genes for each identified pathway is shown in Supplementary Table S4); however, this proved impossible due to the low number of downregulated genes shared by both groups (*n* = 2) and related to C57BL/6 J-only (*n* = 4). Thus, we performed the analysis only on the PrU group. Both algorithms revealed that the genes upregulated by PrUs both in C57BL/6 J and db/db skin were mainly related to ECM organization and interaction (Fig. 7a, a’), a finding also reflected in the group of the genes upregulated in C57BL/6 J only (Fig. 7b, b’). Note that the Alzheimer disease-presenilin pathway identified as the most enriched by the Panther algorithm is related to ECM remodeling (genes included in the pathway: *Mmp9*, *Mmp8*, *Mmp2*, *Mmp13*, *Mmp12*).

On the other hand, specific gene regulation in the db/db group was included in pathways involving inflammation and the immune system (Fig. 7c, c’).

## Discussion

Modeling complex biological processes such as tissue repair to identify potential molecular targets for drug therapy is a challenging task. Modeling skin wound healing requires a consideration of various factors: the different phases of the healing process (coagulation, inflammation, proliferation, and remodeling), and systemic and local conditions which can influence skin vulnerability and repair (such as diabetes), as well as the mechanism of lesion induction, since lesions generated by penetrating trauma (accidental or surgical, defined as “open wounds”) and lesions generated by dermal ischemia (defined as “closed wounds”) are supported by different pathogenetic mechanisms (Masson-Meyers et al. 2020).

In this study, we present a comparative analysis of the two most popular wound models in rodents, the excisional wound and PrU models induced by repeated ischemia–reperfusion cycles (Stadler et al. 2004), to identify the key molecular players related to the lesion type and comorbidities, in this case diabetes. The former model is generated by the surgical removal of all skin layers (epidermis, derma, and subcutaneous fat), while the latter is induced by repeated ischemia–reperfusion cycles applied to the dermal capillary plexus, a key event in the pathogenesis of PrU ulcers in diabetic feet (Mustoe et al. 2006).

In the first part of the study, we adopted the conventional “hypothesis-testing” study design, focusing on sensory innervation and capillary net stabilization at wound closure, while in the second we adopted a “hypothesis-generating” approach, screening the expression level of more than 240 genes driving wound repair, thus investigated at 50% of closure, when the process is active.

The main findings of the study are as follows: (i) the skin in db/db mice shows a dramatic reduction in PGP9.5-IR innervation and a reduced expression of NGF high- and low-affinity receptors compared to C57BL/6 J, such as a reduction of the capillary network and increased expression of the VEGF *Kdr* receptor; (ii) Hifα is strongly upregulated in the PrU model in both genotypes; (iii) the PI3K-Akt signaling pathway is at the core of the altered molecular network of the db/db strain compared to C57BL/6 J mice; (vi) db/db mice show an impairment in the molecular regulation of hypoxia-related genes in the PrU but not in the excision wound (Hif1α, VEGF receptors Flt1, and Kdr); (v) among the investigated genes encoding for ECM, growth factors, and angiogenesis proteins, TGB3, Timp1, FN1, and COL4A1 appear to be the genes upregulated either by hyperglycemia or lesions, indicating the extracellular matrix composition as a landmark which defines genotype and lesion molecular specificity; and (vi) the repair process in db/db mice appears to extend the inflammatory phase.

### Skin biology in diabetic and non-diabetic mice

We included male non-diabetic (C57BL/6 J) and diabetic db/db mice in the study, a widely used mouse model of type 2 diabetes mellitus (Kobayashi et al. 2000; Alpers and Hudkins 2011; Guest and Rahmoune 2019). Db/db mice are homozygous for the diabetes spontaneous mutation (*Lepr*^*db*^) on a C57BL/6 J genetic background, and develop progressive insulin resistance, hyperglycemia, and obesity from 4 to 8 weeks of age. This is followed by progressive sensory loss, electrophysiological impairments, and skin innervation loss (De Gregorio et al. 2018; Giuliani et al. 2020), which stabilize from 16 weeks of age onwards (Shi et al. 2013; Tang et al. 2019), mimicking the main pathophysiological aspects observed in human diabetic neuropathy (Yorek 2016).

Comparative analysis of the two genotypes, as performed by PGP9.5–IR for sensory fibers which visualize the subcutaneous, deep cutaneous, and sub-epidermal fibers, showed a substantial reduction in skin innervation in db/db compared to C57BL/6 J mice. Skin innervation is provided by deep-subcutaneous and subependymal plexus, connected by radial fibers, and by the sub-epidermal plexus supplying the free epidermal nerve endings to the epidermis (Underwood et al. 2001). Since NGF is known to play a key role in establishing sensory innervation of the skin during development and for its maintenance in adulthood (Indo 2010), we analyzed the expression level of the growth factor and related receptors. While NGF mRNA was undetectable in the skin, the mRNA expression level for trkA high- and p75 low-affinity receptors was lower in db/db compared to C57BL/6 J mice, thus suggesting an NGF signaling impairment in the skin of diabetic mice due to the lower receptor expression. NGF receptors in the skin are expressed in keratinocytes, fibroblasts, and endothelial cells (Gostynska et al. 2020), and are present on nerve endings.

We then analyzed the skin capillaries. The vascular architecture of the skin consists of two networks arranged horizontally which are detached from the capillary loops—a lower network between the adipose tissue and derma, and an upper network between the dermis and the epidermis (Huggenberger and Detmar 2011). Capillary density is also dramatically reduced in db/db compared to C57BL/6 J mice in the PrU model. While circulating VEGF is similar in the two genotypes, the down-stream VEGF pathway differs substantially in db/db compared to C57BL/6 J mice. In fact, while a strong upregulation of both VEGF receptors is observed in C57BL/6 J mice, no changes are induced by the ischemia in db/db mice.

Appropriate skin innervation and microcirculation play a preeminent role in wound healing (Okonkwo and Dipietro 2017; Kiya and Kubo 2019). An impairment in NGF signaling may therefore support the “small-fiber neuropathy” described in humans (Sima 2003; Ebenezer and Polydefkis 2014), while a VEGF signaling impairment may affect angiogenesis (Okonkwo et al. 2020), and both of these factors may be responsible for delayed wound healing in diabetic mice.

As far as the “data-driven” approach for the included genes is concerned, we observed a general upregulated profile in db/db compared to C57BL/6 J mice, as indicated by 100 upregulated genes, and only one downregulated gene—Mmp13. This metalloprotease is directly involved in keratinocyte migration and angiogenesis (Hattori et al. 2009) and glucose-mediated neurotoxicity (Waldron et al. 2018), and regulates the gene expression of other Mmps and genes involved in the inflammatory response (Toriseva et al. 2012), highlighting its fundamental role in both diabetes and wound healing.

Moreover, the center molecules of clusters upregulated in db/db are the aforementioned VEGF receptor Kdr, the angiogenesis-related gene Fn1, and the extracellular matrix gene Col4a1. Upregulation of Kdr due to db/db mutation has been described above by real-time PCR analysis, as expected by the induction of its gene by high glucose concentration and its central role in diabetic vasculopathy (Nakagawa et al. 2006). The Fn1 gene and protein are upregulated by high glucose exposure or diabetes (Roy et al. 1990) and in chronic wounds, as already described (Ongenae et al. 2000). Fn1 plasma levels are also higher in diabetic patients, where the gene can be used as a marker for endothelial activation and vascular injury (Kanters et al. 2001). Col4a1 is at the center of a cluster of collagen and other ECM–related proteins. Collagen heterodimerizes with the Col4a2, a gene present in the dysregulated network in db/db tissue, and is the most abundant component of almost all basement membranes (Kuo et al. 2012).

The bioinformatic analysis of the 252 genes included in the study identified the PI3K-Akt pathway as the “core” of the molecular differences between diabetic and non-diabetic skin, thus confirming previously reported results (Huang et al. 2015). Akt1 is essential for the vascular dynamics in wound healing (Somanath et al. 2008), and is activated by growth factors and cytokines, and downstream effectors include glucose, lipid metabolism-regulating molecules, and biological processes such as the inflammatory response, cell migration, proliferation, and apoptosis (Huang et al. 2018). Akt1 is also activated by hypoxia-inducible factors which are required for proper tissue reaction in hypoxia-induced PrU also (Jing et al. 2015). The AKT-pathway is also involved in both NGF and VEGF signaling (Huang et al. 2015; Jere et al. 2019), and TrkA decline in db/db mice may drive AKT-pathway impairment (Vines et al. 2019). It is interesting to note that Mmp13, the only downregulated gene in db/db, appears to exert its pro-healing action through Akt signaling (Toriseva et al. 2012).

AKT-pathway dysfunction in diabetic mice, which has been indicated as a possible cause of wound healing impairment (Huang et al. 2015), is somewhat complex, involving AKT isoform switches, phosphorylation, and de-phosphorylation in the different phases of wound healing (coagulation, inflammation, proliferation, and remodeling) (Maurer et al. 2014; Vines et al. 2019; Jere et al. 2019; Khorami et al. 2020). A transient pharmacological activation of the PI3K-Akt-mTOR signaling axis has been considered a novel clinical intervention strategy to accelerate wound (ulcer) healing (Squarize et al. 2010), and we recently demonstrated that the pro-healing effect of a mutated form of rhNGF includes Akt regulation (Giuliani et al. 2020).

### Wound repair in open and closed lesions

Our study involved the two most widely used skin lesion models. The excision model is performed by skin biopsy needle, removing the epidermis and exposing the derma to the air, while the PrU is induced by repeated ischemia/reperfusion cycles, generating adipose tissue necrosis which extends to the derma and the epidermis without exposing tissues to the air during lesion induction, characterized therefore by tissue hypoxia. A substantial delay in wound repair was observed in diabetic mice in both lesions as expected.

We observed significant differences in lesion histopathology at closure between db/db and C57B/L6 mice according to lesion type, with epidermal hypertrophy being induced by the ischemic PrU but not by the excision wound in both genotypes. It should be stressed that this single time-point study did not include the re-modeling phase, when epidermal hypertrophy is usually reduced.

With regard to skin innervation, no genotype-related differences were observed in either wound model; indeed, the PGP9.5 fiber density was similar to the respective intact control. In both lesion models, however, trkA receptors were upregulated in db/db mice but not in non-diabetic mice. Notably, circulating NGF dramatically rose in the PrU model in db/db mice, possibly related to the severe systemic inflammatory reaction generated by this type of lesion (Jonsjö et al. 2020).

Substantial differences related to wound model were observed in angiogenesis-related gene expression. In the ischemic PrU, the Hif1α expression level rose in both db/db and C57BL/6 J mice, in accordance with the expected tissue reaction to ischemia (Bartoszewski et al. 2019). This increased expression of Hifα1 promotes angiogenesis in open wound models in diabetic mice (Mace et al. 2007; Botusan et al. 2008), but here we demonstrated a severe impairment of downstream molecular regulation in the closed wound model in diabetic mice. While Hif1α positively regulates the expression of several genes, including VEGF, this is followed by a VEGF receptor increase in non-diabetic mice, which does not occur in diabetic mice. Due to the upregulation of *Kdr* gene expression in intact skin of db/db mice, we can argue that the downstream signaling is impaired. 3-Kinase-Akt pathway (PI3K) is an important VEGF signaling pathway mediated by KDR, and the altered pAkt/Akt ratio observed in diabetic skin could finally result in a reduced PECAM-positive capillary net as observed in db/db PrU. In fact, the *Kdr* VEGF receptor is one of the node genes differentiating db/db and C57BL/6 J mice.

Minor changes were observed in the excision model in both genotypes. Thus, the molecular signaling activated by wounds differs according to systemic diseases, such as diabetes, as well as to lesion type, particularly in closed as opposed to open lesions.

As far as the “data-driven” approach for the included genes is concerned, we found different centers in the activated gene expression networks in the active phases of the wound repair, according to genotype and lesion type. In non-diabetic mice, MMP9 and AKT are at the center of activated genes in the excision model, while ITGB3, Fn1, and MMP9 are at the center of the PrU model.

In db/db mice, Timp1 and MMP9 are at the core of the PrU model, while Timp1 is at the core of the excision model. Mmp9 is a main effector of the ulcer response, and as shown in the PrU network, it corresponds to an increase of Timp1 and Timp2, the two main inhibitors of metalloproteinases. An equilibrium between Mmps and Timps is fundamental for ulcer healing, and the Mmp9/Timp1 serum level ratio has been shown to be an indicator of healing, with increased levels of Mmp9 predicting poor wound healing in human diabetic patients (Li et al. 2013).

There is a sharp contrast between the Mmp9 and Timp1 ratios of the two ulcer models in C57BL/6 J mice. Skin excision evoked a strong upregulation of Mmp9 (fold of change: 29.90) compared to Timp1 (fold of change: 4.66), while the PrU produced a lower upregulation of Mmp9 (fold of change: 19.45) and a considerable increase in Timp1 expression (fold of change: 39.01). In db/db animals on the other hand, skin excision evoked a low Mmp9 gene expression increase (fold of change: 3.15) and Timp1 upregulation (fold of change: 11.92), while the PrU resulted in a dramatic increase in Mmp9 (fold of increase: 45.37) with less Timp1 activation (fold of increase: 28.95).

Fibronectin encoded by the Fn1 gene is also in the upregulated network in non-diabetic mice, but not in db/db mice. Fibronectin is a fundamental component of the ECM and a key player in wound healing, being involved at all stages of the process (Lenselink 2015), and has even been proposed as a therapeutic tool (Wang et al. 2019). However, here we showed that this gene is highly expressed in the db/db strain at basal level (fold of change: 8.85), upregulated only by the PrU and only in C57BL/6 J mice (fold of change compared to intact: 12.27).

The pathway enrichment analysis on PrUs in diabetic and non-diabetic mice also showed that the PrU evokes different responses, with an imbalance towards an inflammatory reaction in db/db mice. As already mentioned, the healing process is generated by different consecutive and partially overlapping steps: homeostasis, inflammation, proliferation, and remodeling (Wang and Xu 2020). Thus, the transcriptomic analysis 14 days after PrU formation revealed that the db/db wound appears to have stalled in the inflammation phase, while the ulcerated skin of the C57BL/6 J animals has already moved forward to the proliferation/remodeling step.

These results corroborate the PrU experimental lesion as the most suitable for mimicking the wound healing process in diabetic models and for testing new treatments; indeed, inflammation is a fundamental player in the pathogenic conditions triggered by diabetes, and is regarded as a key therapeutic target (Tsalamandris et al. 2019). Together with infections, unresolved inflammation is a hallmark of diabetic non-healing wounds (Santra et al. 2020), and topical treatments are proposed to promote healing, mainly by reducing inflammation (Wang and Xu 2020).

## Conclusions

In this study, we demonstrated that the molecular network activated by skin wounds differs according to underlying systemic diseases (e.g., diabetes) and to lesion type (excision vs pressure), and these two factors should be carefully considered when testing new drug candidates. We believe that the search for potential new pharmacological targets should take advantage of the large amount of data we gathered from the “hypothesis-generating” omics approach. The major findings of our study reflect the principles of the Working Group on the Diabetic Foot (WGDF), which highlight the variability of the vascular and neuropathic components of diabetic foot ulcers, and the importance of investigating the molecular pathology of the lesion (Rayman et al. 2020).

## Supplementary information

Below is the link to the electronic supplementary material.

Supplementary file1 (XLSX 23 KB)

Supplementary file2 (XLSX 19 KB)

Supplementary file3 (XLSX 15 KB)

Supplementary file4 (XLSX 42 KB)
